# The Diagnostic Role of Computed Tomography for ACR TI-RADS 4–5 Thyroid Nodules With Coarse Calcifications

**DOI:** 10.3389/fonc.2020.00911

**Published:** 2020-06-05

**Authors:** Peiying Wei, Niandong Jiang, Jinwang Ding, JingJing Xiang, Luoyu Wang, Haibin Wang, Ying Gu, DingCun Luo, Zhijiang Han

**Affiliations:** ^1^Department of Radiology, Affiliated Hangzhou First People's Hospital, Zhejiang University School of Medicine, Hangzhou, China; ^2^Department of Radiology, Chunan County Hospital of Traditional Chinese Medicine, Hangzhou, China; ^3^Department of Surgical Oncology, Affiliated Hangzhou First People's Hospital, Zhejiang University School of Medicine, Hangzhou, China; ^4^Department of Pathology, Affiliated Hangzhou First People's Hospital, Zhejiang University School of Medicine, Hangzhou, China; ^5^Department of Psychology, Institute of Psychological Sciences, Hangzhou Normal University, Hangzhou, China; ^6^Department of Ultrasonography, Affiliated Hangzhou First People's Hospital, Zhejiang University School of Medicine, Hangzhou, China

**Keywords:** thyroid nodule, coarse calcification, ACR TI-RADS, computed tomography, ultrasonography

## Abstract

**Objectives:** Coarse calcifications are prone to cause echo attenuation during ultrasonography (US) and hence affect the classification of benign and malignant nodules. This study aimed to investigate the diagnostic role of computed tomography (CT) for differentiating the American College of Radiology (ACR) Thyroid Imaging Reporting and Data System (TI-RADS) 4–5 nodules with coarse calcifications.

**Methods:** CT data of 216 ACR TI-RADS 4–5 nodules with coarse calcifications confirmed by surgery and pathology in 207 patients were analyzed retrospectively. Halo sign, artifacts, and CT values (i.e., Hounsfield unit) of the nodules were determined by two radiologists. Univariate analysis and binary logistic regression were used to determine the relationship of halo sign, artifact, and CT value with benign nodules. A predictive model for benign nodules with coarse calcifications was then constructed. The receiver operating characteristic (ROC) curve was used to analyze the predictive value of halo sign, artifact, CT value, and logistic regression model.

**Results:** Of the 216 ACR TI-RADS 4–5 nodules with coarse calcifications, 170 were benign and 46 were malignant. There were 92 benign and 7 malignant nodules with halo sign (χ^2^ = 22.067, *P* < 0.001), and 79 benign and 10 malignant nodules with artifacts (χ^2^ = 9.140, *P* < 0.001). The CT values of benign and malignant nodules were 791 (543–1,025) Hu and 486 (406–670) Hu, respectively (*Z* = −5.394, *P* < 0.001). Binary logistic regression demonstrated that the halo sign, artifact, and CT value were independent predictors for benign nodules with coarse calcifications. The area under the ROC curve (AUC) of halo sign, artifact, CT value and regression model for predicting benign nodules with coarse calcifications were 0.776, 0.711, 0.784, and 0.850, respectively, and the optimal threshold of CT value was 627.5 Hu.

**Conclusion:** Halo sign, artifact, and CT value > 627.5 Hu were helpful for identifying ACR TI-RADS 4–5 thyroid benign nodules with coarse calcifications. The diagnostic performance of the logistic regression model was higher than that of any single indicator. Accurate identification of these indicators could identify benign nodules and reduce unnecessary surgical trauma.

## Introduction

Calcification is a common imaging finding observed in thyroid nodules. It accounts for 15.7–28.9% of benign nodules and 49.6–65.9% of malignant nodules in ultrasonography (US) (1, 2). The diagnostic value of microcalcification for malignant nodules has been widely accepted (1–3). However, coarse calcifications often cause obvious echo attenuation, which affects the visualization of internal and posterior structures of the calcified nodules. Moreover, coarse calcifications are often accompanied by fibrosis in the surrounding regions, which results in uneven echo and fails to provide enough information for a proper diagnosis. Therefore, thyroid nodules with coarse calcification are usually classified as the American College of Radiology (ACR) Thyroid Imaging Reporting and Data System (TI-RADS) 4 or 5 (4–6), and patients are recommended to undergo fine-needle aspiration biopsy (FNAB) (7). FNAB is considered the gold standard for the diagnosis of most thyroid nodules (8, 9). However, for thyroid nodules with coarse calcifications, especially those with thick-walled annular calcifications, it is difficult to penetrate the hard calcified regions or obtain enough sample for cytology, even if the needle could penetrate the hard calcified area (9, 10). This results in a failure rate of 30–70% (11, 12).

Although computed tomography (CT) is inadequate for the diagnosis of microscopic and diffuse lesions in the thyroid, it is able to fully reveal the internal and surrounding structures of ACR TI-RADS 4 or 5 nodules with coarse calcifications. This is due to the non-appearance of echo attenuation. In our previous studies, we analyzed solitary coarse calcified nodules using the artifact and CT histogram independently. We found that both the presence of artifacts around calcifications on unenhanced CT and the proportion of voxels < 1,150 Hu not more than 98.4% could contribute to the diagnosis of benign nodules (13, 14). Evaluating artifact is relatively simple, however, the operation of CT histogram is more complicated and requires specialized analytical software. We believe that the CT value (i.e., Hounsfield unit) could also reflect the structural characteristics inside calcifications to a certain extent. In addition, for ACR TI-RADS 4 or 5 coarse calcified nodules that contain soft tissues, enhanced CT could reflect blood supply of the soft tissues and hence determine their properties.

Based on our previous studies (13–17), this study aimed to combine the features of unenhanced and enhanced CT to determine the diagnostic role for ACR TI-RADS 4–5 thyroid nodules with coarse calcifications. This could provide an important basis for further diagnosis and treatment in the clinic.

## Materials and Methods

### Patient Data

From August 2017 to August 2019, data of eligible patients were retrospectively analyzed. The inclusion criteria were as follows: (1) ACR TI-RADS 4 or 5 thyroid nodules; (2) Complete data obtained using unenhanced and enhanced CT scans; (3) The short diameter of the calcified area ≥ 4 mm; and (4) Pathological verification after surgery. The exclusion criteria were as follows: (1) nodules that were not clearly visible on CT images due to image quality or other thyroid diseases; and (2) patients with incomplete clinical and imaging data. Using the Picture Archiving and Communication System (PACS) workstation, the keywords “calcification” or “high echo” were used to search for eligible cases. Finally, a total of 216 coarse calcified nodules (207 patients) were obtained, of which, there were 154 and 62 nodules characterized as ACR-TIRADS 4 and 5, respectively, and 170 nodules (163 patients) were benign and 46 nodules (44 patients) were malignant. All patients provided written informed consent for study participation. This study was approved by the local Ethics Committee of the Affiliated Hangzhou First People's Hospital, Zhejiang University School of Medicine. The study was performed in accordance with the Declaration of Helsinki.

### CT Examination

All patients underwent unenhanced and enhanced CT scans using a 16-slice spiral CT scanner (Lightspeed, GE, United States). Patients were placed in the supine position and scanned from the base of the skull to the upper edge of the aortic arch. The scan parameters were as follows: 120 kV, 250 mA, 0.625 × l6 mm collimation, 0.938 pitch, 0.5 s rotation time, 3.75 mm cross-sectional thickness, and 3.75 mm cross-sectional distance. The contrast used was 60 ml Iopromide Injection (Bayer, Germany). Iodine concentration was 370 mg/mL and the contrast was injected through the elbow vein using a high-pressure syringe at a rate of 2.0–2.5 mL/s. The enhanced scans were initiated at 50 s after contrast injection.

### Image Analysis

**Figure 1:**
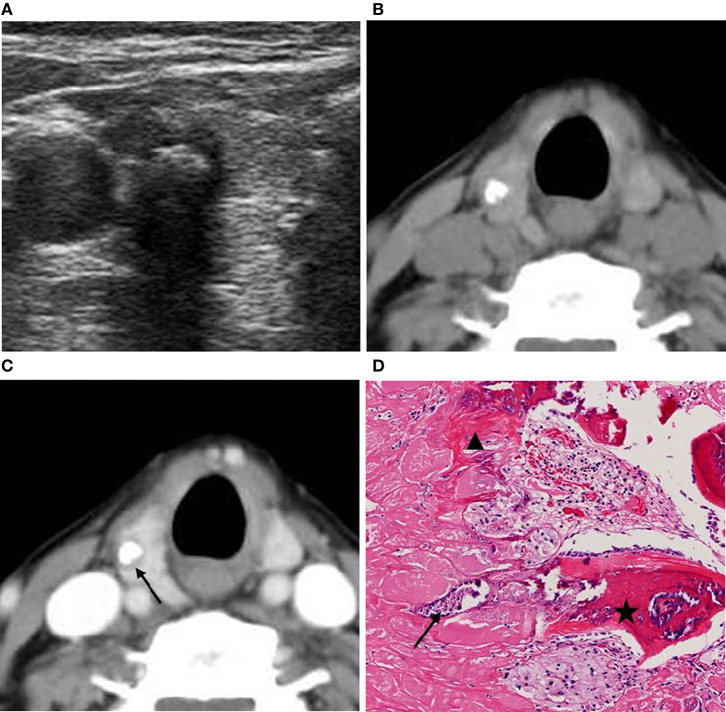
A 63-year-old woman with a coarse calcified nodule on the right lobe of the thyroid. **(A)** US classified the nodule as ACR TI-RADS 5, and showed significant echo attenuation in the posterior region of the calcified nodule. **(B)** Unenhanced CT showed no low-density region around the calcified nodule. **(C)** Enhanced CT showed zonal low-density regions around the calcified nodule. **(D)** Pathological image displayed (H&E × 80 magnification) that calcifications (black arrow) and fibrosis (black triangle) coexisted and were accompanied with ossifications (black pentagram). The lesion was pathologically confirmed as NG.

**Figure 2:**
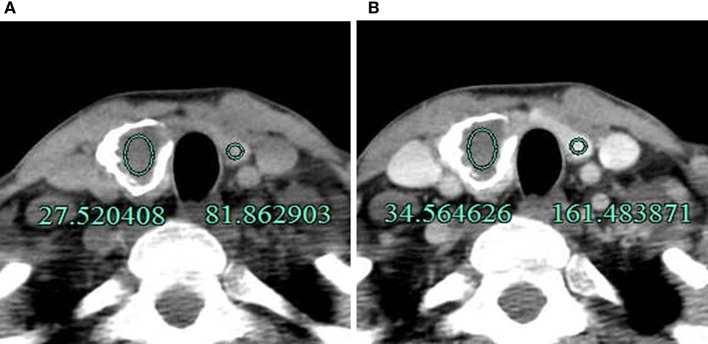
A 40-year-old man with an annular calcified nodule on the right lobe of the thyroid. **(A)** The difference in CT value between the thyroid tissue and inside the calcified nodule was 54.34 Hu (81.86–27.52 Hu) on unenhanced CT. **(B)** The difference after enhancement was 126.92 Hu (161.48–34.56 Hu) and was higher compared to unenhanced CT. The lesion was pathologically confirmed as NG.

**Figure 3:**
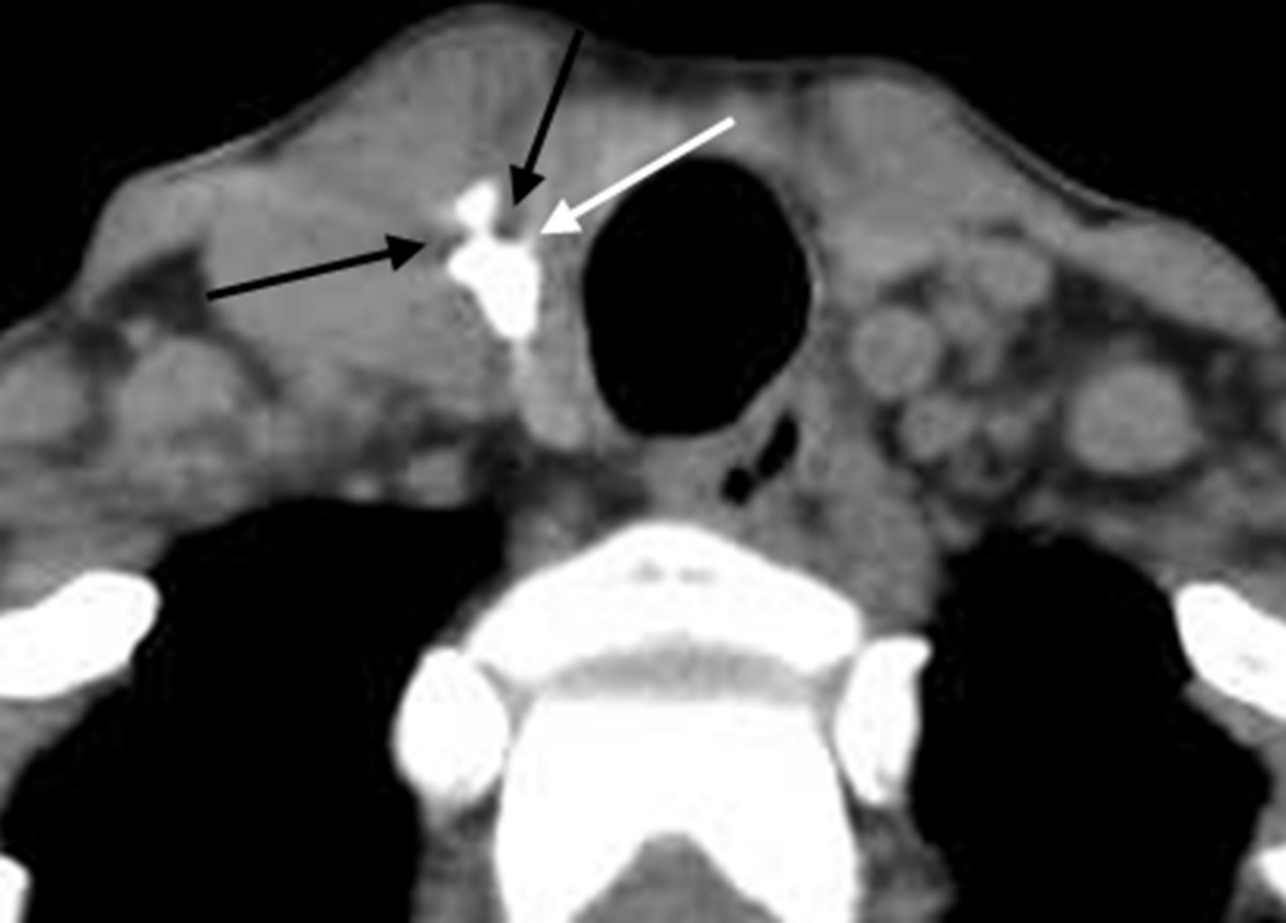
Unenhanced CT showed obvious cupping artifacts (white and black arrow). The lesion was pathologically confirmed as NG.

**Figure 4:**
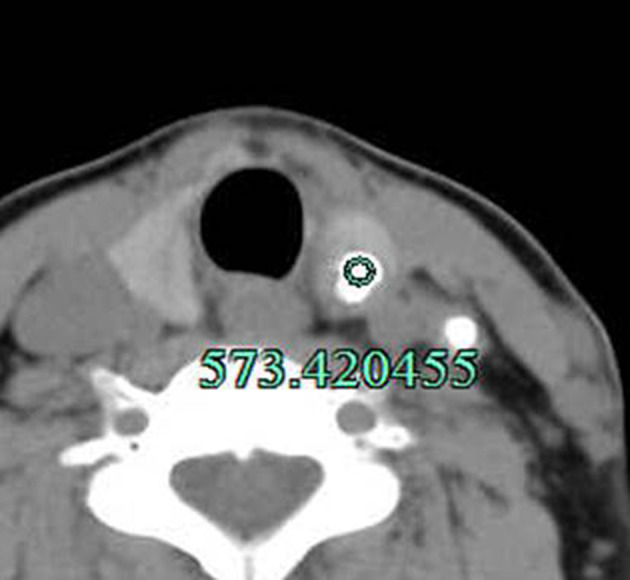
Method for measuring the CT value of the calcified s in nodule. The lesion was surgically confirmed as PTC. In addition, lymph node metastasis with calcification in the left IV group was observed.

CT images were read independently by two radiologists who had 5 and 8 years of working experience, respectively. The radiologists were blinded to the clinical information, other imaging results, and pathological data of the patients. They evaluated the halo sign and artifacts around nodules and measured the CT values of nodules. Halo sign was defined as that the area around or inside calcified nodules become evident in enhanced CT compared to unenhanced CT (Figure 1), i.e., the difference in CT value between the thyroid tissue and the area around the calcification or within the annular calcification nodule in enhanced CT was greater than that in unenhanced CT (Figure 2). CT artifacts were cupping artifacts or the appearance of dark bands or streaks between the dense objects in the image (13, 18) (Figure 3). The CT value of the calcified area was measured under soft tissue window width (WW) and window position (WL) (WW: 350, WL: 40). A circular or elliptical area was selected as the region of interest (ROI), which should be as large as possible. Afterwards, the CT values in the maximum and sub-maximal sections of the calcified areas were measured. Non-calcified areas within the ROI was avoided (Figure 4). If there were any discrepancies in determining halo sign and artifact between the two radiologists, a final decision was made after a consensus was reached. The average values measured by the two radiologists were taken as the final value of CT values.

### Pathology

All 216 ACR TI-RADS 4–5 thyroid nodules from 207 patients had a conclusive pathological diagnosis confirmed by senior pathologists. Archived tissue specimens were made into five μm-thick sections and fixed with 10% neutral formalin and stained using the hematoxylin/eosin (H&E) method. The tissue sections were then examined under a light microscope.

### Statistical Analysis

Statistical analyses were performed using the SPSS 22.0 software and LibSVM library toolbox 3.23 in Matrix Laboratory. Data with normal distribution were expressed as mean ± standard deviation, while data with non-normal distribution were expressed as median (interquartile rang). Classification data were analyzed using the chi-square test or the Fisher exact test, while continuous variables were compared using the independent-samples *t*-test. Continuous variables were tested using the independent-samples *t*-test or the Wilcoxon rank sum test. Multivariate analysis were performed using binary logistic regression. The diagnostic performances was assessed by the receiver operating characteristic (ROC) curve (AUC), sensitivity, specificity, and accuracy. Cohen kappa coefficient was used to assess the consistency between two radiologists. A difference with *P* < 0.05 was considered statistically significant.

## Results

### General Characteristics

**Figure 5:**
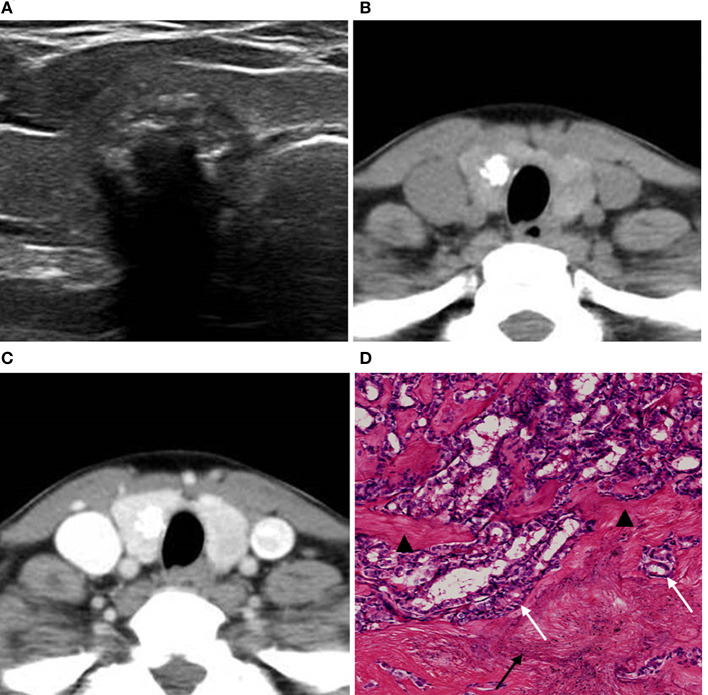
A 49-year-old woman with a coarse calcified nodule on the right lobe of the thyroid. **(A)** US classified the nodule as ACR TI-RADS 5, and showed multiple imbricate calcifications inside the nodule with obvious echo attenuation. **(B)** Unenhanced CT showed no artifact around the calcifications. **(C)** Enhanced CT showed no halo sign around the calcification. **(D)** Pathological image displayed (H&E × 80 magnification) that calcifications (black arrow) and fibrosis (black triangle) separated by cancer tissues (white arrow). The lesion was surgically confirmed as PTC.

All 170 benign thyroid nodules from 163 patients were nodular goiter (NG) (Figures 1–3), while 45 of the malignant nodules from 44 patients were papillary thyroid carcinoma (PTC) (Figures 4, 5) and 1 nodule was follicular carcinoma. The malignancy rates were approximately 11.7 and 45.2% for ACR-TIRADS 4 and 5, respectively. There were 25 males and 138 females with benign nodules and 11 males and 33 females with malignant nodules (*P* = 0.133). The mean age of male patients was 55.95 ± 12.14 years and for females was 53.48 ± 11.02 years (*P* = 0.707). No significant difference was found between the patients with benign nodules and malignant nodules in any relevant thyroid hormone levels (Table 1). Among all the patients, the triiodothyronine (T3), free triiodothyronine (FT3), free thyroxine (FT4) levels were normal. However, there were 6 (3.7%) patients with benign nodules and 2 (4.5%) with malignant nodules showing abnormal thyroxine (T4) levels (*P* = 0.792), 20 (12.3%) patients with benign nodules and 2 (4.5%) with malignant nodules showing abnormal TSH (thyroid-stimulating hormone) levels (*P* = 0.140), respectively.

**Table 1:** The general characteristics and CT features distribution.

**Variable**	**Benign**	**Malignant**	***P*-value**
**Sex**, ***N*** **(%)**			
Male	25 (15.24)	11 (25.00)	0.133
Female	138 (84.76)	33 (75.00)	
Age, years	55.95 ± 12.14	53.48 ± 11.02	0.707
Total T3, ng/dL	1.03 (0.91–1.12)	1.04 ± 0.20	0.911
Total T4, ng/dL	92.58 ± 19.67	88.01 ± 17.19	0.256
Free T3, ng/dL	4.77 (4.46–5.11)	4.83 (4.54–5.3)	0.150
Free T4, ng/dL	16.72 ± 2.25	16.42 ± 2.80	0.577
TSH, μIU/L	2.54 ± 4.80	2.16 ± 2.06	0.681
Size, mm	10.90 (7.43–14.70)	8.70 (6.80–13.0)	0.069
**Halo sign**, ***N*** **(%)**			
Yes	92 (54.12)	7 (15.22)	<0.001
No	78 (45.89)	39 (84.78)	
**Artifact**, ***N*** **(%)**			
Yes	79 (43.89)	10 (21.74)	<0.001
No	91 (56.11)	36 (78.26)	
CT value, Hu	791 (543–1025)	486 (406–670)	<0.001

*Values are expressed as number (%), mean ± standard deviation, or median (interquartile range). T3, triiodothyronine; T4, thyroxine; TSH, thyroid-stimulating hormone*.

### CT Features of ACR TI-RADS 4–5 Benign Nodules

The average lesion size of benign and malignant nodules was 10.90 (7.43–14.70) mm and 8.70 (6.80–13.0) mm, respectively (*Z* = −1.817, *P* = 0.069). There were 92 benign and 7 malignant nodules with halo sign (χ^2^ = 22.067, *P* < 0.001), and 79 benign and 10 malignant nodules with artifacts (χ^2^ = 9.140, *P* < 0.001). The CT value for benign nodules was 791 (543–1,025) Hu and for malignant nodules was 486 (406–670) Hu (*Z* = −5.394, *P* < 0.001) (Table 1).

### Logistic Regression Model Construction

Binary logistic regression was performed by considering halo sign, artifact, and CT value as independent variables, and benign thyroid nodules with coarse calcification as the dependent variable. Results demonstrated that halo sign, artifact, and CT value were independent predictive factors for benign thyroid nodules with coarse calcifications (Table 3). Based on the multivariate analysis results, a logistic regression model was constructed: Logistic (*P*) = −2.368 + 2.324 × halo sign + 0.912 × artifact + 0.004 × CT value (Table 2).

**Table 2:** Binary logistic regression analysis for CT features of benign nodules with coarse calcifications.

**Risk factors**	***B***	**SE**	**Walds**	***P***	**OR**	**95%CI**
Halo sign	2.324	0.484	23.047	<0.001	10.214	3.955–26.376
Artifact	0.912	0.454	4.033	0.045	2.489	1.022–6.060
CT value	0.004	0.001	17.794	<0.001	1.004	1.002–1.006
Constant	−2.368	0.626	14.287	<0.001	0.094	

### Diagnostic Performances for Benign Nodules With Coarse Calcifications

**Figure 6:**
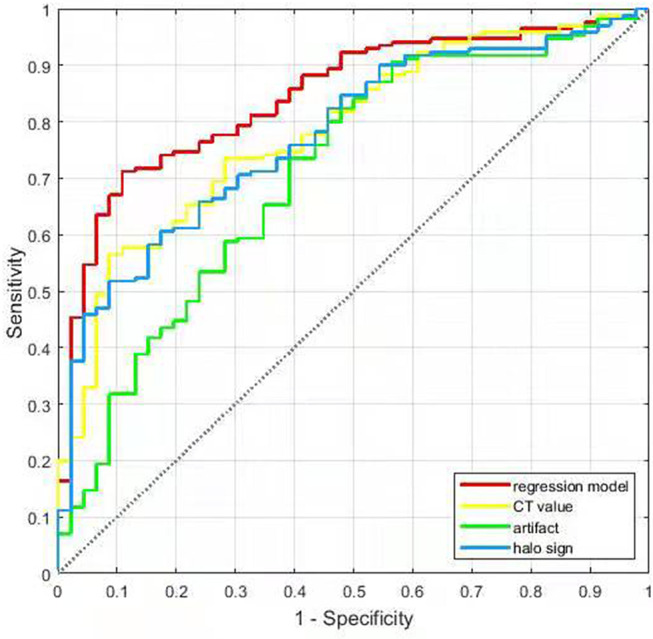
The ROC curves of the logistic regression model and each single indicator.

For predicting the benign nodules with coarse calcifications, The AUCs for halo sign, artifact, CT value, and regression model were 0.776, 0.711, 0.784, and 0.850, with sensitivities of 54.1, 46.5, 67.7, and 65.3%, specificities of 84.8, 78.3, 73.9, and 91.3%, accuracies of 60.7, 53.2, 69.0, and 70.8%, respectively, and the optimal threshold of CT value was 627.5 Hu (Table 3 and Figure 6).

**Table 3:** Comparison of diagnostic performances for benign thyroid nodules with coarse calcifications.

**Predictors**	**AUC**	**Sensitivity**	**Specificity**	**Accuracy**
Halo sign	0.776	54.1%	84.8%	60.7%
Artifact	0.711	46.5%	78.3%	53.2%
CT value	0.784	67.7%	73.9%	69.0%
Regression model	0.850	65.3%	91.3%	70.8%

### Interobserver Consistency Analysis

The two radiologists had good agreement on halo sign (*k* = 0.889; 95%CI: 0.824–0.944), artifact (*k* = 0.896; 95%CI: 0.833–0.953), and CT value (*k* = 0.840; 95%CI: 0.738–0.925).

## Discussion

In our previous studies, we exclusively used the artifact and CT histogram to analyze the features of thyroid solitary coarse calcified nodules (13, 14). Based on these findings, we used conventional neck CT scan and proposed the halo sign for nodule classification for the first time. Combining the halo sign with artifact and CT value CT, we were able to differentiate benign and malignant ACR TI-RADS 4–5 nodules with coarse calcifications. Our results demonstrated that in benign nodules with coarse calcifications, halo sign, and artifacts were more common, and the CT values were significantly higher compared to malignant nodules. Halo sign, artifact, and CT value were independent predictors for benign nodules with coarse calcifications. In addition, the diagnostic performance of the constructed logistic regression model was superior compared to any single indicator.

At present, the use of CT for thyroid nodules with coarse calcifications have been rarely reported. Publications have mainly focused on unenhanced CT for analyzing the morphology and density of calcifications (19, 20), while a limited number of studies have been published on the use of enhanced CT. Yang et al. (21) analyzed 41 cases of solitary calcified nodules and 55 cases of coarse calcified nodules. They found that enlarged lesions observed using enhanced CT was an independent risk factor for cancer. However, the method, WW, and WL for measuring ROI were not addressed, and the lesion size was not quantified in their study. Our results demonstrated that halo sign after enhancement was an independent risk predictor for the diagnosis of benign nodules with coarse calcifications. In addition, the odds ratio of halo sign was significantly higher than that of artifact and CT value. We infer the mechanism is that the thyroid tissue is significantly enhanced, while fibrotic tissue, which has lower or no blood supply around the calcification or within annular calcification nodule, has mild or no enhancement. Hence, the density difference between the area around the calcification or within the annular calcification and the thyroid tissue is increased compared to unenhanced CT. This results in halo sign. However, the majority of fibrotic tissue that is present around the calcification or within annular calcification in malignant nodules are invaded and segmented by tumor tissue and hence have increased blood supply (22). This results in higher enhancement. Hence, the density difference between the area around calcification or within annular calcification and the thyroid tissue is decreased, which results in no halo sign.

Several studies have been performed to investigate the density of thyroid nodules with coarse calcifications. Holtz and Powers (23) had observed in 1958 that the density of calcifications in benign thyroid nodules was higher compared to that in malignant nodules using X-ray examinations. However, they failed to perform further quantitative studies due to technical difficulties. Kim et al. (19) demonstrated that the CT values of benign calcified nodules were higher compared to malignant nodules, however they did not further analyze its diagnostic efficacy due to their small sample cohort. In our study, we found that both artifact and CT value were independent predictors for the diagnosis of benign coarse calcified nodules. Artifact and CT value have the same pathological mechanism. Most of the benign calcifications or ossifications are continuous and manifest as dense plates, blocks, strips, arcs, or are nodular in shape. They are associated with abundant accumulation of calcium salts. However, malignant coarse calcifications are often interrupted with malignant tumor cell infiltration, which manifest as loose plaques or have punctate distribution. It is related to the insufficient accumulation of calcium salts and the continuous destruction by malignant tumor cells during the accumulation process (13, 24). Judging artifact is simple and relatively intuitive, however, the measurement of CT value can be objective and quantified. The diagnostic efficacy of CT value (AUC = 0.784) was higher compared to artifact (AUC = 0.711).

The results of our study confirmed that the diagnostic value of halo sign on enhanced CT was much more than two other signs on unenhanced CT. However, there may be a concern regarding the use of iodinated contrast agents, which can compete with ^131^I and interfere with subsequent postoperative radioiodine (RAI) therapy (25, 26). It should be noted that the recommendations against performing enhanced CT seem to be based on studies conducted in the past when lipophilic contrast agents were in use, which tend to get stored in adipose tissues for long time (27, 28). Whereas currently majority of centers use water-soluble ionic contrast agents, which are unlikely to be retained in extracellular fluids (29). Therefore, the urinary iodine concentration (UIC) should soon revert back to previous equilibrium after enhanced CT. Many studies also showed that the time for UIC to normalize was 30–43 days (29–32). In routine practice, the time for patients from finishing enhanced CT examination to starting RAI therapy is far more than 43 days. Moreover, the majority of studies showed that the timing of post-thyroidectomy initial RAI therapy did not affect the overall survival or long-term outcomes (33–38). Therefore, RAI administration may be safely planned according to the logistics of the local health and the patient itself. Besides, we should also not deter from requesting enhanced CT, since it has a great many advantages in terms of proper mapping, staging, planning of surgery, and preparedness for additional help if required.

In the present study, the malignancy rates is ~11.7 and 45.2% for ACR-TIRADS 4 and 5 respectively, which were lower than some previous studies reported (the malignancy rates of 14.5–49.6% and 81.4–92.9% for ACR-TIRADS 4 and 5, respectively) (7, 39, 40). We considered this was related to the fact that the nodules we included were all coarse calcified nodules characterized as ACR-TIRADS 4 and 5 by ultrasound. According to the 2017 ACR-TIRADS white paper (4), in nodules with calcifications that cause strong acoustic shadowing that precludes or limits assessment of internal characteristics, particularly echogenicity and composition, it is best to assume that the nodule is solid and assign two points for composition and one point for echogenicity. That is to say, coarse calcifications may increase the ACR-TIRADS scores to a certain extent, thus many coarse calcified nodules that were actually at low risk were categorized as ACR-TIRADS 4 or 5. Therefore, it is of great significance for us to identify the benign coarse calcified nodules that are easily over-categorized by ultrasound, since it can reduce unnecessary surgical trauma and waste of medical resources.

There are several limitations to this study. First, this was a retrospective study and the data was obtained from a single medical institute, which may lead to a certain degree of selection bias. Second, the number of malignant coarse calcified nodules was small. Additional multicenter studies using larger patient cohorts are necessary to validate our findings. Finally, considering the layer thickness for CT scans was 3.75 mm, to avoid the influence of partial volume effect, we did not include the ACR TI-RADS 4–5 nodules with a short diameter of calcified region < 4 mm. Hence, whether our results could be applied to these nodules needs to be determined.

In conclusion, halo sign, artifact, and CT value > 627.5 Hu are helpful for classifying ACR TI-RADS 4–5 benign thyroid nodules with coarse calcifications. The diagnostic efficacy of our logistic regression model was higher compared to any single indicator investigated in this study. Accurate measurement of these indicators could identify benign nodules and reduce unnecessary surgery. In addition, it is simple to operate, highly repeatable, and easily transferable.

## Data Availability Statement

The datasets generated for this study are available on request to the corresponding author.

## Ethics Statement

The studies involving human participants were reviewed and approved by Hangzhou First People's Hospital. Written informed consent to participate in this study was provided by the participants' legal guardian/next of kin.

## Author Contributions

PW, NJ, DL, and ZH conceived and designed the study. JD and HW collected the clinical and image data of all cases. JX provided pathological results. PW, NJ, and YG reviewed, analyzed, and classified the imaging data. PW, JD, and LW performed statistical analysis. PW and NJ wrote the first draft of the manuscript. All authors contributed to manuscript revision and approved the submitted version.

## Conflict of Interest

The authors declare that the research was conducted in the absence of any commercial or financial relationships that could be construed as a potential conflict of interest.
